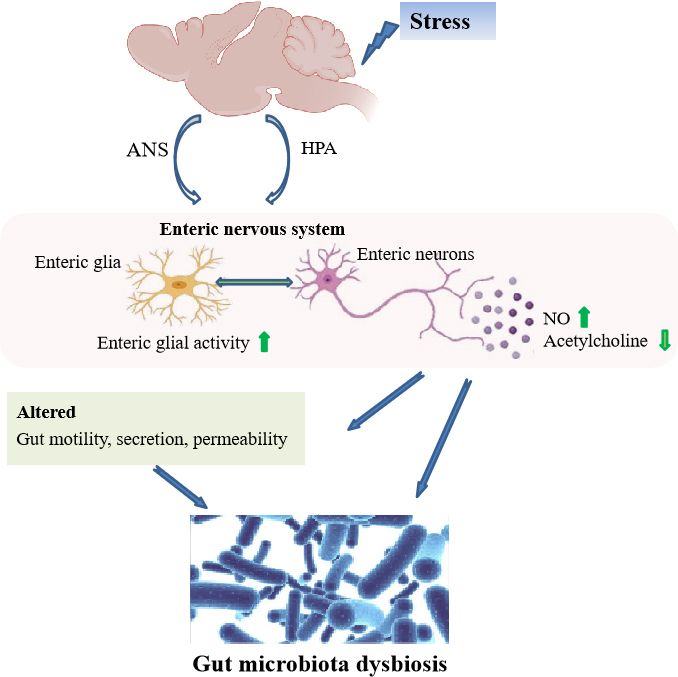# Enteric glial cells contribute to chronic stress-induced alterations in the intestinal microbiota and barrier in rats

**DOI:** 10.1016/j.heliyon.2024.e24899

**Published:** 2024-01-23

**Authors:** Tong Lu, Chenxu Huang, Rongxin Weng, Zepeng Wang, Haiji Sun, Xiaoli Ma

**Affiliations:** a Shandong Intelligent Technology Innovation Center, Central Hospital Affiliated to Shandong First Medical University, Jinan, 250013, China; b Key Laboratory of Animal Resistance Biology of Shandong Province, School of Life Science, Shandong Normal University, 88^#^ Wenhua Road, Jinan, 250014, China

**Keywords:** Intestinal microbiota, Enteric glial cells, Water-avoidance stress, Enteric nervous system

## Abstract

**Background:**

Emerging evidence has demonstrated the impact of psychological stress on intestinal microbiota, however, the precise mechanisms are not fully understood. Enteric glia, a unique type of peripheral glia found within the enteric nervous system (ENS), play an active role in enteric neural circuits and have profound effects on gut functions. In the present study, we tested the hypothesis that enteric glia are involved in the alterations in the intestinal microflora and barrier induced by chronic water-avoidance stress (WAS) in the gut.

**Methods and results:**

Western blotting and immunohistochemical (IHC) staining were used to examine the expression of glial fibrillary acidic protein (GFAP), nitric oxide synthetase (NOS) and choline acety1transferase (ChAT) in colon tissues. 16S rDNA sequencing was performed to analyse the composition of the intestinal microbiota in rats. Changes in the tight junction proteins Occludin, Claudin1 and proliferating cell nuclear antigen (PCNA) in the colon tissues were detected after WAS. The abundance of Firmicutes, Proteobacteria, Lactobacillus and Lachnospiraceae_NK4A136 decreased significantly, whereas the abundance of Actinobacteria, Ruminococcaceae_UCG−005 and Christensenellaceae-R-7 increased significantly in stressed rats. Meanwhile, the expression of Occludin, Claudin1 and PCNA significantly decreased after WAS. Treatment with L-A-aminohexanedioic acid (L-AA), a gliotoxin that blunts astrocytic function, obviously decreased the abundance of Actinobacteria, Ruminococcaceae_UCG−005 and Christensenel-laceae_R-7 in stressed rats and significantly increased the abundance of Proteobacteria, Lactobacillus and Lachnospiraceae_NK4A136. In addition, the protein expression of colon Occludin, Claudin1, and PCNA increased after intraperitoneal injection of L-AA. Furthermore, the expression level of NOS in colon tissues was significantly decreased, whereas that of ChAT was significantly increased following L-AA treatment.

**Conclusions:**

Our results showed that enteric glial cells may contribute to WAS-induced changes in the intestinal microbiota and barrier function by modulating the activity of NOS and cholinergic neurones in the ENS.

## Abbreviations

WAS: Water avoidance stress
GFAP: Glial fibrillary acidic protein
NOS: NO synthetase
ChAT: Choline acety1transferase
PCNA: Proliferating cell nuclear antigen
L-AA: L-A-aminohexanedioic acid
GI: Gastrointestinal
IBS: Irritable bowel syndrome
HPA: Hypothalamic–pituitary–adrenal
ENS: Enteric nervous system
NAFLD: Nonalcoholic fatty liver disease
NO: Nitric oxide
VIP: Vasoactive intestinal peptide
GAT2: GABA transporter 2

## 1 Introduction

Stress is the general response of the body to physiological, physical or psychological stimuli that disrupts homeostasis. The gastrointestinal (GI) tract is a primary target of stress, and stress has been shown to have profound effects on the GI tract, including alterations in intestinal motility [1], mucosal transport [2], gut barrier function [3], and visceral sensitivity [4]. Moreover, these alterations may cause stress-related functional GI disorders, such as irritable bowel syndrome (IBS) [5] and peptic ulcers [6]. Recent evidence suggests that stress exposure can signiﬁcantly change gut microbial populations. The abundance of microbiota residing in the human intestine was estimated to be 10^14^ microorganisms. The gut microbiotas can prevent infections caused by pathogens, provide nutrients, such as vitamins B and K, contribute to the digestion and absorption of macromolecules, shape the mucosal immune system, and serve as a biological barrier [[7], [8], [9]]. Additionally, preclinical evidence suggests that the microbiota and its metabolites are likely to be involved in modulating behaviours and brain processes, including emotional behaviour [10], pain modulation [11] and digestive behaviour [12]. The intestinal microbiota is important for maintaining health, and disruption in the establishment of a stable, normal gut microbiota may be associated with or even contribute to the pathogenesis of diseases, including inflammatory bowel disease, nosocomial infection, and neonatal necrotising enterocolitis [13].

A growing body of evidence has revealed that stress exposure in the early postnatal period, infancy, or adulthood can change the composition of the host gut microbiota and that the gut microbiota can influence behaviours, including those relevant to anxiety and depression [14]. Adult mice exposed to a social disruption stressor showed an altered gut microbiota and increased circulating cytokine levels [15]. This stress led to a decrease in the abundance of *Limosilactobacillus reuteri*, an immunomodulatory bacterial species. It has also been shown that both acute stress and repeated stress affect the levels of intestinal secretory immunoglobulin (Ig) A, impacting intestinal homeostasis, the inﬂammatory response and possibly dysbiosis [16]. Various microbiota-targeted interventions can ameliorate chronic stress-induced deﬁcits in mice, which include the administration of live bacterial strains (probiotics), as well as the supplementation of host-indigestible dietary ﬁbres that are fermented by the gut microbiota (prebiotics). For example, stress-induced changes in the hypothalamic-pituitary-adrenal (HPA) axis and autonomic nervous system display sensitivity to probiotic intervention (*Lactobacillus helveticus* R0052 and *B*iﬁdo*bacterium longum* R0175). These probiotics also restored the integrity of colonic tight junctions in stressed mice [17]. Probiotics induce bacterial adhesion and translocation to the mesenteric lymph nodes in response to stress [18]. Investigators have used faecal microbiota transplantation approaches to demonstrate that stress-related microbiota composition plays a causal role in behavioural changes. In one example, investigators showed that transplanting the microbiota from stressor-exposed conventional mice to GF mice resulted in exaggerated inﬂammatory responses to *Citrobacter rodentium* infection [19]. A link between disease-related microbiota and behaviors was recently demonstrated, in which faecal microbiota transplantation from depressed patients to microbiota-depleted rats increased anhedonia and anxiety-like behaviors [20]. Collectively, these results reveal that stress results in an altered gut microbial composition. Changes in the stress-related microbiota may play a critical role in the pathophysiology of stress-related disorders. However, the mechanism underlying the effect of stress on the gut microbiota remains unclear.

The enteric nervous system (ENS) consists of millions of neurones and glial cells organised into interconnected ganglia embedded within the gut wall. The major functions of the ENS that have been most studied include the regulation of local gut motility, secretion, and blood flow. Recent studies have shown that the physiological functions of the GI tract can also affect the gut microbiota. Gut motility is recognised as the primary factor controlling microbial levels in the length of the GI tract [21]. Pharmacological manipulation of GI motility is associated with altered microbial populations [22]. Impaired intestinal transit caused by compromised, migrating motor complexes is associated with bacterial overgrowth in the small intestine [23]. In addition, some microbes, such as members of the genus *Lactobacillus*, contain mucous-binding proteins that help them bind to the intestinal mucous layer [24,25]. Thus, changes in mucous secretions have the potential to change microbial populations. Convergent reports suggest a profound role of the ENS in modulating microbiota composition. Thus, it is possible that under stress conditions, the ENS is stimulated, and along with the integrity of the GI epithelial barrier, gut secretions and motility are altered, leading to the disruption of the gut microbiota. The aim of this study was to examine the changes in the gut microbiota of rats following exposure to chronic water-avoidance stress (WAS) and the role of the ENS, including enteric glial cells, in stress-induced gut microbiota alterations.

## 2 Results

### 2.1 The effects of WAS on the intestinal microbiota in rats

**Fig. 1:**
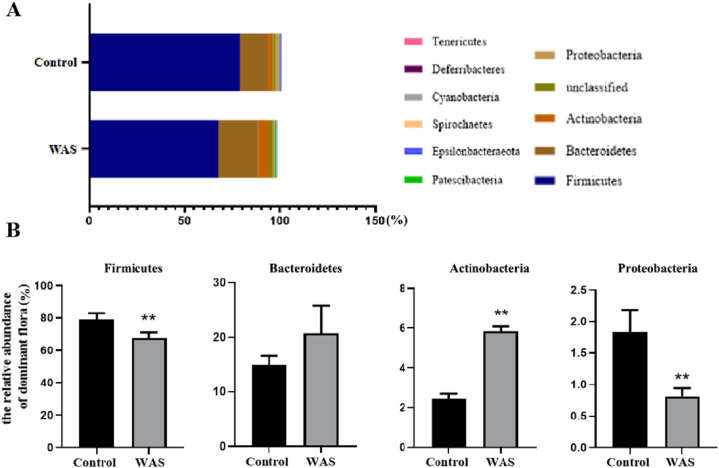
Relative abundance of dominant intestinal flora at the phylum level in rats (A) Stacked histogram of the relative abundance of dominant flora. (B) Circular chart of the relative distribution proportion of dominant flora: Firmicutes, Bacteroidetes, Actinobacteria and Proteobacteria. Control group. Data are presented as the mean ± standard error of the mean (SEM). One-way ANOVA was used. *P < 0.05, **P < 0.01 vs. control group (n = 5/group).

At the phylum level, 16S rRNA gene sequencing data illustrated that 11 different phyla were identified across all faecal samples (Fig. 1A), of which only four had more than 1 % overall relative abundance: Firmicutes, Bacteroidetes, Actinobacteria and Proteobacteria. Phylum-level analysis showed that the relative abundances of Firmicutes and Proteobacteria were significantly decreased, whereas the relative abundances of Actinobacteria was significantly higher in the WAS group than in the control group (Fig. 1B). However, no difference in the relative abundance of Bacteroidetes was observed between the control and WAS groups.

**Fig. 2:**
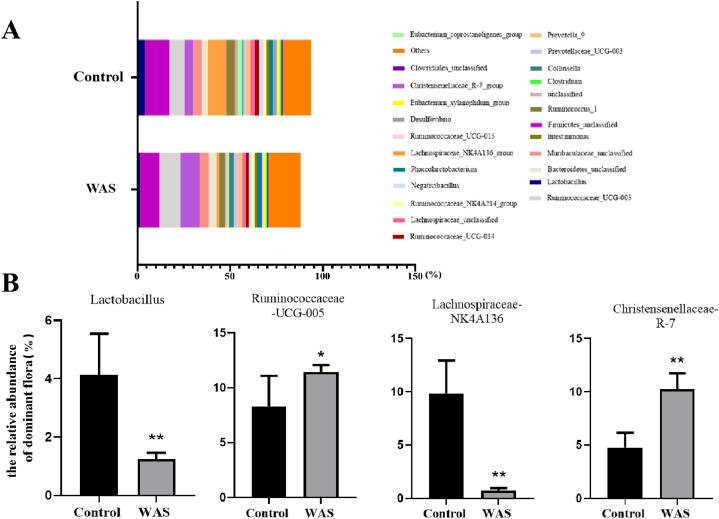
Relative abundance of dominant intestinal flora at the genus level in rats (A) Stacked histogram of the relative abundance of dominant flora. (B) Circe chart of the relative distribution proportion of dominant flora: *Lactobacillus,* Ruminococcaceae_UCG−005, Lachnospiraceae_ NK4A136_group, and Christensenellaceae_R-7_group. Data are presented as the mean ± SEM. One-way ANOVA was used. *P < 0.05, **P < 0.01 vs. control group (n = 5/group).

At the genus level, different bacteria were identified in the faecal samples (Fig. 2A). The abundance of *Ruminococcus_UCG_005* and Christensenellaceae_R_7 significantly increased, whereas that of *Lactobacillus* and Lachnospiraceae_NK4A136 significantly decreased after WAS in rats (Fig. 2B).

### 2.2 Intestinal barrier protein and glial fibrillary acidic protein (GFAP) expression in rats with WAS

**Fig. 3:**
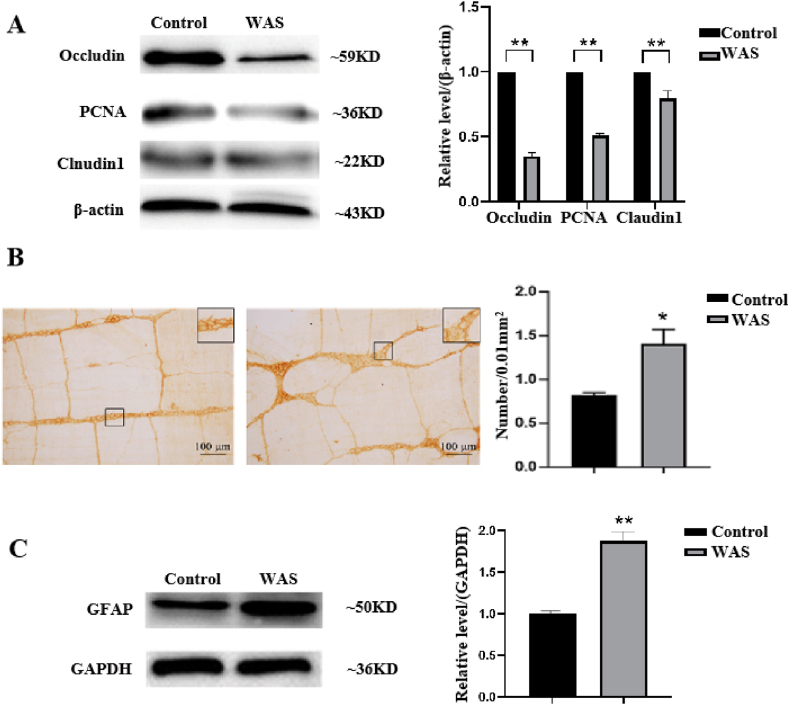
Expression of intestinal barrier protein and GFAP in rats with WAS. (A) Expression of Occludin, PCNA, and Claudin1; (B) The number of enteric glial cells in the myenteric nerve plexus of rats/0.01 mm^2^ (n = 5/group). (C) Expression of GFAP proteins in the colon tissue of rats treated with WAS. Quantiﬁcation of the relative average grayscale value of GFAP/GAPDH (n = 5/group). Data are presented as the mean ± SEM. One-way ANOVA was used. *P < 0.05, **P < 0.01 vs. control group.

To further explore the effects of chronic WAS on the intestinal barrier function, the expression of tight junction proteins such as Occludin, Claudin1 and proliferating cell nuclear antigen (PCNA), was determined using western blotting. The results indicated that the expression of Occludin, Claudin1, and PCNA was significantly decreased in the colon tissue of the WAS group compared to that in the control group (*p* < 0.05; Fig. 3A). PCNA is expressed in the nuclei of cells during the DNA synthesis phase of the cell cycle and is involved in the proliferation of the intestinal epithelium. PCNA may play an important role in the damage and repair of the intestinal mucosa [26]. GFAP is a commonly used marker to identify enteric glia in the mammalian gut [27]. To investigate whether enteric glia in the myenteric plexus of the colon are involved in WAS, we determined the number of GFAP-positive enteric glial cells in the myenteric plexus of the colon using immunohistochemical (IHC) staining using a whole-mount preparation technique and western blotting. The number of GFAP-positive enteric glial cells was significantly higher in the treatment group than that in the control group (Fig. 3B). Western blotting results were consistent with the IHC staining results (Fig. 3C). These results suggested that enteric glia are involved in the process of WAS.

### 2.3 Effect of L-A-aminohexanedioic acid (L-AA) on intestinal flora in rats with WAS

**Fig. 4:**
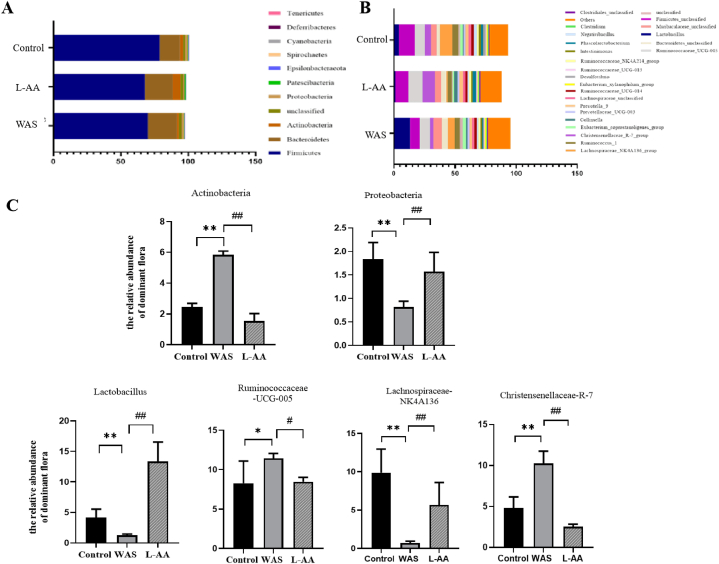
Relative abundance of dominant intestinal flora in rats. (A) Stacked histogram of relative abundance at the phylum level of dominant flora. (B) Stacked histogram of relative abundance at the genus level of dominant flora. (C) Abundance of Actinobacteria, Proteobacteria, *Lactobacillus,* Ruminococcaceae_UCG−005, Lachnospiraceae_NK4A136, and Christensenellaceae_R-7_group. *P < 0.05, **P < 0.01 control group.

Similar to astrocytes in the CNS, EGCs contain GFAP [28]. To explore the role of enteric glial cells in the changes in the intestinal microbiota induced by WAS, L-AA, a gliotoxin that blunts astrocytic function, was used. L-AA is a glutamate analogue that acts as a competitive inhibitor of glutamine synthetase [29], and induces a transient dysfunction of astrocytes in vivo by entering astroglial cells through Na^+^-dependent glutamate transporters and inhibiting cellular functions including protein synthesis and metabolic processes [30]. Recent studies have shown that L-AA reduces astrocytic GFAP and glutamate aspartate transporter (GLAST) expression and ATP-linked mitochondrial respiration in astrocytes, but does not affect microglial Iba1^+^ cell counts or expression of the macrophage reactivity marker cluster of differentiation 68 (CD68), and does not affect neuronal viability or neuronal complexity in vitro and in vivo, thus indicating a selective action of L-AA on astrocytes [31]. Compared with the WAS control group, the relative abundances of the Actinobacteria phylum and Christensenellaceae_R_7 significantly were decreased in the WAS treated with L-AA group, whereas the relative abundances of Proteobacteria, Lachnospiraceae_NK4A136, and *Lactobacillus* were significantly increased (Fig. 4A–C). However, no significant differences were observed in the relative abundance of Ruminococcaceae _UCG_005 as compared to the saline-treated WAS group.

### 2.4 Effects of L-AA on intestinal barrier proteins in rats with WAS

**Fig. 5:**
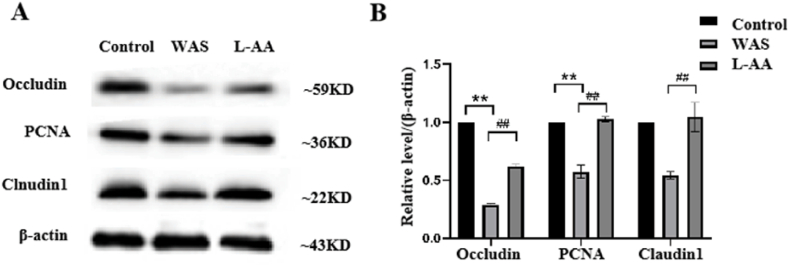
Expression of Occludin, Claudin1 and PCNA proteins in rats. (A) Expression of Occludin, Claudin1 and PCNA proteins in the colon tissue of rats treated with L-AA. (B) Quantiﬁcation of the relative average grayscale value of Occludin, Claudin1 and PCNA proteins.

Gut permeability is mainly determined by the integrity of paracellular tight junctions, which include transmembrane junctional proteins such as Occludin and Claudins [32]. Compared with rats with WAS treated with saline, rats with WAS treated with L-AA (marked as LAA in the figure) showed significantly increased protein expression of Occludin, Claudin1 and PCNA in the colon tissue (Fig. 5A and B). These results indicate that L-AA could increase intestinal barrier proteins and prevent the impairment of intestinal barrier function induced by WAS.

Effects of L-AA on NO synthetase (NOS) and choline acety1transferase (ChAT) in the ENS in rats with WAS.

**Fig. 6:**
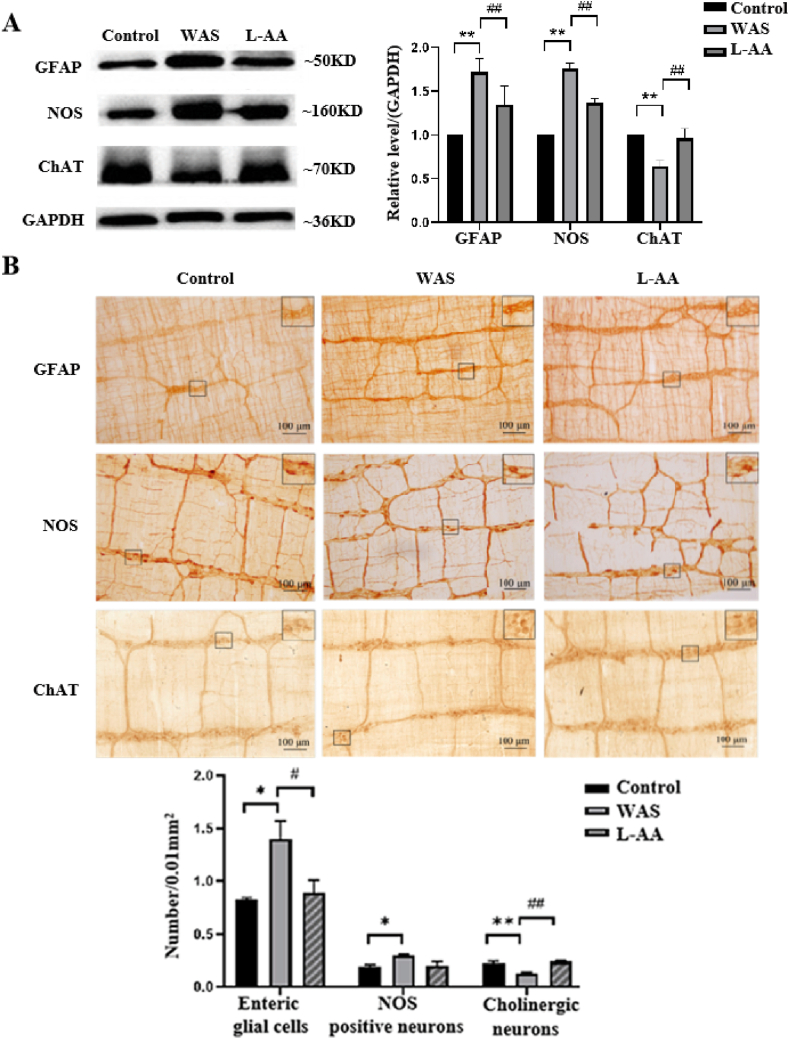
Expression of GFAP, NOS, and ChAT proteins in the colon tissue of rats. (A) Western blotting revealed the expression of GFAP, NOS, and ChAT proteins in rat colon tissues. Quantiﬁcation of the relative average grayscale values of Occludin, Claudin1, and PCNA/GAPDH (n = 5/group). (B) IHC staining of the number of enteric glial cells, NOS-positive neurones and cholinergic neurones in the myenteric nerve plexus of the rats/0.01 mm2 **P < 0.01vs. control group; ^#^*P* < 0.05，^##^*P* < 0.01 vs. WAS group.

The expression of GFAP, NOS and ChAT in the ENS was detected using western blotting. The results showed that pretreatment with L-AA markedly attenuated GFAP protein expression induced by WAS (*P* < 0.05) (Fig. 6A). Furthermore, compared with rats with WAS, treatment with L-AA led to a significant increase in NOS protein expression (*P* < 0.01), whereas ChAT protein expression was significantly decreased (*P* < 0.05) (Fig. 6A). The IHC staining results were similar to the western blotting results (Fig. 6B). These results suggested that L-AA decreased the number of enteric glial cells. Furthermore, NO neuron activity was enhanced, and cholinergic neuron activity was attenuated in the colonic ENS of rats exposed to WAS.

## 3 Discussion

The intestinal microbiota is a large and complex microbial community containing approximately 1000 known bacterial species, belonging to six major phyla: Firmicutes, Bacteroidetes, Proteobacteria, Actinomycetes, Verrucomicrobia, and *Fusobacteria* [33]. The intestinal microbiota can regulate the nutrient supply [34], prevent pathogen colonisation, and shape and maintaining host immunity [35]. In addition, gut microbiota can regulate gut movement, the gut barrier system, and fat distribution. The intricate relationship governing host-gut microbiota interactions suggests that when this relationship is abnormal, the intestinal microbiota may cause disease pathogenesis or promote disease progression. For example, changes in the gut microbiota contribute to the development of metabolic diseases such as obesity, type II diabetes, and nonalcoholic fatty liver disease (NAFLD) [36].

Stress is a systemic nonspecific adaptive response that occurs when the body is stimulated by internal and external environmental factors, and leads to anxiety, fear, elevated blood glucose, increased blood pressure, increased heart rate, and accelerated respiration [37]. Several studies have shown that stress can disrupt the intestinal microbiota. Adult mice subjected to chronic restraint stress showed a significant decrease in caecum flora abundance and diversity [38]. Chronic social defeat stress can lead to a significant reduction in caecum flora diversity and the relative abundance of Bacteroidetes and *Lactobacillus* in mice, and an increase in the relative abundance of *Fusobacterium*. Mice exposed to chronic unpredictable mild stress have a lower relative abundance of *Lactobacillus* and a higher relative abundance of *Akkermansia* in their intestine [3]. Chronic WAS in rats is extensively employed as a reproducible and robust model of chronic psychological stress, with minimal physical stress, to better mirror the experience of ongoing environmental and life stress in humans [39]. In the present study, we found that the gut microbiota composition of rats subjected to WAS was signiﬁcantly altered compared with that of control rats. At the phylum level, the abundances of Firmicutes and Proteobacteria significantly decreased in rats with WAS, whereas the abundance of Actinobacteria significantly increased. Furthermore, the abundances of the genera Rumenococcaceae-UCG-005 and Christensenellaceae-R-7 were significantly increased in rats with WAS, whereas the abundances of *Lactobacillus* and Lachnospiraceae-NK4A136 were significantly decreased. These results are consistent with studies reporting that chronic psychological stress results in the outgrowth of *Akkermansia* and decreases the abundance of Firmicutes [40]. In addition, another study found that psychological stress decreased the abundance of Bacteroides, Alistipes, and *Lactobacillus* and increased the abundance of Parasutterella and Rikenellaceae_RC9_gut_group in stressed mice [1]. Therefore, it is possible that different stress models have different effects on the gut microbiota.

The intestinal barrier is the major barrier that separates the body from the external environment and limits the permeation of harmful luminal molecules while allowing the appropriate absorption of nutrients and water. Gut permeability is primarily determined by the integrity of paracellular tight junctions, which include transmembrane junctional proteins such as Occludin and Claudins [32]. Chronic stress induces enhanced intestinal epithelial permeability, which is related to changes in tight junction proteins within the epithelium [41].

Here, the protein levels of Occludin, and Claudin1 in the colon were significantly decreased in rats with WAS compared to those in the control group, suggesting that WAS affected mucosal barrier integrity and led to intestinal barrier dysfunction. These results are consistent with previous reports that psychological stress generally increases intestinal permeability, regardless of the type of stress, such as communication box-induced stress or restraint stress [42,43]. PCNA is an intranuclear protein that assists DNase. PCNA has no specificity for species, genera, or tissues, and is present in actively proliferating cells [44]. A previous study showed that exposure to heat stress for 24 h significantly decreased the number of PCNA-positive cells in the duodenum and increased plasma endotoxin concentrations, suggesting that heat stress directly affects intestinal morphology and permeability in chickens exposed to heat stress for 24 h [45]. The current study demonstrated that the expression of PCNA signiﬁcantly decreased in the colon tissue of rats after WAS when compared with that of the control group, suggesting that chronic stress can decrease the proliferation ability of epithelial cells. Low proliferative ability leads to a low ability to repair the intestinal mucosa and may be associated with dysfunction of the mucosal barrier.

The ENS is composed of enteric neurones and enteric glial cells that interact with different cells, including muscle cells, epithelial cells, interstitial cells of Cajal (ICCs), blood vessels, and immune cells [46], and regulates the smooth muscle, mucosal epithelium, and vascular effector systems in the GI tract [47]. It is now well established that gut microbes and their released factors can communicate with the ENS through direct and indirect actions on enteric neurones and enteric glial cells. The intestinal microbiota and its metabolites have been shown to be involved in modulating GI functions and affecting intestinal permeability [48], intestinal motility [49], and activity in the ENS [50]. In a recent study, stress-induced alterations in ENS activity were inﬂuenced by both maternal separation and the microbiota, thus providing evidence that the intestinal microbiota may underpin ENS-related gut dysfunction associated with early-life stress [51]. Although microbes can affect the neural activity in the ENS, the ENS seemingly controls the microbiota. In a mouse model of colorectal aganglionosis, signiﬁcant differences in microbiota diversity over time were observed and were characterised by increasing diversity in mutant aganglionic mice. These studies suggested that ENS may contributed to WAS-induced changes in microbiota.

Enteric glia are a large population of peripheral neuroglia are associated with the cell bodies and processes of enteric neurones throughout the digestive tract. Enteric glial cells can regulate intestinal epithelial barrier function [52], modulate neurotransmitters through transporter proteins [53], participate in functional GI responses, and may play a role in GI motility abnormalities [54]. Recent studies have shown that enteric glial cells not only play a key role in the ENS, intestinal health, and functional homeostasis, but also have a close link with the intestinal microbiota [55]. In the present study, we found that enteric glial cells were activated under chronic WAS. Furthermore, inhibition of enteric glial cells with L-AA resulted in a significant decrease in the abundance of the Actinobacteria phylum, A. tumefaciens_UCG-005, and C. christensenii_R-7, and a significant increase in the abundance of the *Aspergillus phylum, Lactobacillus* and Trichosporon_NK4A136, compared with the chronic WAS group, indicating that enteric glial cells are involved in chronic WAS-induced disruption of the rat intestinal microbiota. In addition, our findings showed that the protein expression of Occludin, Claudin1, and PCNA was significantly decreased in the colon tissue following chronic WAS. However, L-AA treatment significantly increased the protein levels of Occludin, Claudin1 and PCNA in the colon tissue of rats undergoing stress, suggesting that enteric glial cells may be activated by WAS and involved in the stress-induced intestinal barrier dysfunction. These observations are similar to the results of conditional genetic ablation of enteric glial cells in mice, which results in the disruption of intestinal integrity and a profound loss of intestinal barrier function [56]. Destruction of the enteric glial network by chemicals results in the collapse of the epithelial lining, and delayed mucosal healing [57]. These observations highlight the involvement of enteric glial cells in the regulation of gut barrier function.

Functional data have shown that bidirectional communication between enteric glia and neurones regulates enteric reflexes and innervates the digestive tract. Excitatory and inhibitory motor neurones innervate smooth muscle cells and the muscularis mucosae of the GI tract through the release of neurotransmitters. The major neurotransmitters released by excitatory neurons are acetylcholine and substance P, whereas inhibitory neurotransmitters include nitric oxide (NO) and vasoactive intestinal peptide (VIP) [58]. Repeated acute stress (RASt) increased the proportion of ChAT-immunoreactive neurons [59] and acute stress could activate cholinergic myenteric neurones [60,61]. In this study, we found a decrease in myenteric cholinergic neurones in rats treated with chronic stress, which is consistent with the results of cholinergic neurones in the myenteric plexus of the ENS of the rat colon under chronic stress [62]. Changes in the cholinergic neurones induced by acute or chronic stress have not been directly addressed and requires further research.

Chronic stress induced by the WAS protocol increased the number of myenteric cholinergic neurones. In the present study, we observed a decrease in the number of myenteric cholinergic neurones in WAS-treated rats. Enteric glia regulate neurotransmitter availability through their expression of transporters such as GABA transporter 2 (GAT2), and by degrading neuroactive compounds in the extracellular space through cell-surface enzymes, including nucleoside triphosphate diphosphohydrolase. In our study, we found that L-AA treatment decreased the release of the inhibitory neurotransmitter NO and increased the secretion of the excitatory neurotransmitter acetylcholine in WAS rats. Therefore, it is likely that WAS activates enteric glial cells, which may affect the release of neurotransmitters and the activity of enteric neurones, leading to intestinal microbiota disorders. These findings are in line with the previous studies that constructed mutants with abnormal development of the ENS using zebrafish as a model and found that individuals with abnormal development of the ENS had a significant increase in the abundance of pro-inflammatory bacteria and a decrease in the abundance of anti-inflammatory bacteria in the gut, suggesting that the ENS maintains intestinal health by regulating the composition of the intestinal microbiota and gut barrier function [63].

## 4 Conclusions

We conclude that enteric glial cells may contribute to WAS-induced changes in the intestinal microbiota and barrier proteins by modulating the activity of NOS and cholinergic neurones in the ENS (Supplement Fig. 1). The present study has certain limitations. First, WAS may alter colon transit, which in and of itself, may cause changes in the microbiota, however, this has not been considered in the results. Secondly, the mechanisms of enteric glial cells in chronic stress-induced alterations in the intestinal microbiota and barriers have not been clarified. Third, changes in other intestinal segments require further research.

## 5 Methods

### 5.1 Animals

Adult male Wistar rats weighing 240–260 g were obtained from the Shandong University Experimental Animal Centre. Rats were housed under standard conditions at of 21 ± 1 °C and 55 ± 10 % humidity under a 12-h light/dark cycle (lights on at 07.00 h). Food and water were provided ad libitum throughout the study. All experiments were conducted according to the guidelines of the International Association for the Study of Pain (Zimmermann, 1986) and approved by the Experimental Animal Ethical Association of Shandong Normal University(Number of the Animal Experimentation Ethics Committee: AEECSDNU 2021023).

WAS.

The rats were randomly divided into the following three groups of six animals each: control, WAS and L-AA inhibition. The L-AA inhibition groups were administered an intraperitoneal injection of L-AA (98 % l-α-aminoadipic acid and saline preparation, 1.2 mg/mL) for 10 days consecutively, whereas the control and rats with WAS were administered equal volumes of saline. The rats treated with WAS were placed on a square platform (7 × 7 × 9 cm) mounted in the centre of a white transparent plastic container (40 × 50 × 32 cm), which was filled with room temperature fresh tap water (25 °C) to 1 cm below the surface of the platform. The rats were exposed to this paradigm for 1 h each day for 10 days consecutively. The control rats were placed in a similar cage without water for 1 h. The basic data of effects of chronic water avoidance stress on body weight, fecal pellet output (FPO) and visceral sensitivity in rats were provided as supplement data (Supplement Fig. 2).

DNA extraction, polymerase chain reaction (PCR) amplification of 16S rRNA genes, and MiSeq sequencing.

To profile the microbial composition, total genomic DNA was extracted from faecal samples using the QIAamp DNA Stool Mini Kit, following the manufacturer's instructions (QIAGEN, Hilden, Germany). DNA concentration and purity were determined using a Nanodrop 2000 (Thermo Fisher Scientific, Waltham, MA, USA), and DNA integrity was determined by 1 % agar-gel electrophoresis. The V3–V4 regions of 16S rRNA were amplified using PCR with the forward primer 338F (5′-ACTCCTACGGGAGGCAGCG-3′). An Illumina MiSeq platform was used for next-generation sequencing of the PCR products from the amplification of 16S rRNA genes. Sequencing services were provided by Tiny Gene Bio-Tech Co., Ltd. (Shanghai, China). Raw sequencing data were provided in the FASTQ format for downstream analysis. UniFrac distances and community similarity and diversity were computed from sequence data using Quantitative Insights into Microbial Ecology (QIIME) version 1.9.1. Taxonomic data were generated at each rank level (Phylum, Class, Order, Family, Genus) to develop a composite microbial profile. The proportions of each taxonomic classification in each sample were calculated, as a percentage of the total microbial community.

## 6 IHC

Segments of the colon were removed and washed with 0.1 M phosphate-buffered saline (PBS) and subsequently fixed with 4 % paraformaldehyde in 0.1 M PBS for 6–8 h and stored in PBS at 4 °C. After fixation, samples were opened along the mesenteric border. The mucous membrane, submucosa and circular muscle were peeled back to produce whole-mount preparations of the colonic myenteric plexus. The tissue was stretched and pinned on balsa wood with the mucosal side down, and then fixed in 4 % formaldehyde in 0.1 M phosphate buffer (PB), pH 7.0, at 4 °C for 1 h. Whole-mount preparations were washed with TBS three times and incubated in PBS containing 10 % normal goat serum for 1 h to reduce nonspecific background staining. Tissues were incubated for 24 h at 4 °C in a solution containing anti-GFAP (1:500, Chemicon, Temecula, CA, USA), anti-NOS (1:500, Boster, China) and anti-ChAT (1:300, Proteintech, CHI, USA) primary antibodies. Next, the specimens were washed thrice for 10 min in PBS, followed by incubation for 1 h in a buffer solution containing affinity-purified secondary anti-rabbit IgG antibody (1:2000, ZSGB-BIO, China). Finally, the specimens were washed with PBS (3 × 5 min) and cover-slipped with PBS containing 80 % glycerol.

## 7 Western blotting

Protein extracts were prepared as previously described. Following Sodium dodecyl-sulphate polyacrylamide gel electrophoresis (SDS-PAGE), the gel were cut based on the locations of the corresponding protein bands. Proteins were then transferred to polyvinylidene difluoride (PVDF) membranes and incubated with the following primary antibodies separately overnight at 4 °C: rabbit anti-GAPDH (1:1000 Goodhere Biotechnology, China), anti-GFAP (1:1000, Chemicon, Temecula, CA, USA), anti-PCNA(1:2000, Cell Signalling Technology, Danvers, MA, USA, anti-claudin1 (1:1000, HUABIO, China), anti-β-actin (1:50000, Genetex, Irvine, CA, USA), anti-NOS (1:500, Boster, China), and anti-ChAT antibody (1:300, Proteintech, CHI, USA). Subsequently, the membranes were incubated with secondary antibodies for 1 h at room temperature. Chemiluminescence detection methods were used to examine the blots, and the optical density (OD) of the bands was calculated using Quantity One. GAPDH or β-actin served as an internal control protein in the western blotting quantification in the manuscript.

### 7.1 Statistical analysis

The data were statistically analysed using SPSS 25.0 software, with independent samples *t*-test and one-way analysis of variance (ANOVA) test, and the results were expressed as the mean ± standard deviation (‾x ± s), with statistically significant differences set at *P* < 0.05 and highly statistically significant difference set at *P* < 0.01. The Omic Share Tools platform and GraphPad Prism software (version 8.0) were used to plot the results.

## Funding

This research was funded by the 10.13039/501100001809National Natural Science Foundation of China (No.: 32170496).

## Data availability statement

Data will be made available on request.

## CRediT authorship contribution statement

**Tong Lu:** Writing – original draft, Project administration, Methodology, Investigation. **Chenxu Huang:** Writing – original draft, Project administration, Methodology, Investigation. **Rongxin Weng:** Investigation. **Zepeng Wang:** Methodology, Investigation. **Haiji Sun:** Writing – review & editing, Project administration, Funding acquisition, Conceptualization. **Xiaoli Ma:** Writing – review & editing, Project administration, Investigation, Conceptualization.

## Declaration of competing interest

The authors declare that they have no known competing financial interests or personal relationships that could have appeared to influence the work reported in this paper.

## Figures and Tables

**figs1:**