# Supplementary material for: Enteric glial cells contribute to chronic stress-induced alterations in the intestinal microbiota and barrier in rats
Source: Heliyon. 2024 Jan 23;10(3):e24899. doi: 10.1016/j.heliyon.2024.e24899 (PMC10838753; doi:10.1016/j.heliyon.2024.e24899)
Supplement: Multimedia component 1 [file mmc1.docx]

In order to test whether the water avoidance stress model has an effect on the body weight of rats, we selected rats with an initial body weight of 300g for the experiment. We recorded the weight changes during the molding period daily. The rats who avoided water stress for 10 days lost weight compared with the control group (Suppl. Fig 1a). During the experiment, fecal pellet output (FPO) number was significantly higher in stressed rats than in the sham group (Suppl. Fig1 b). In mechanical colorectal distention (CRD) tests, chronic water avoidance stress significantly improved Abdominal withdrawal reflex (AWR) scores in rats stressed by 40 and 80 mmHg (Suppl. Fig1. c).

**
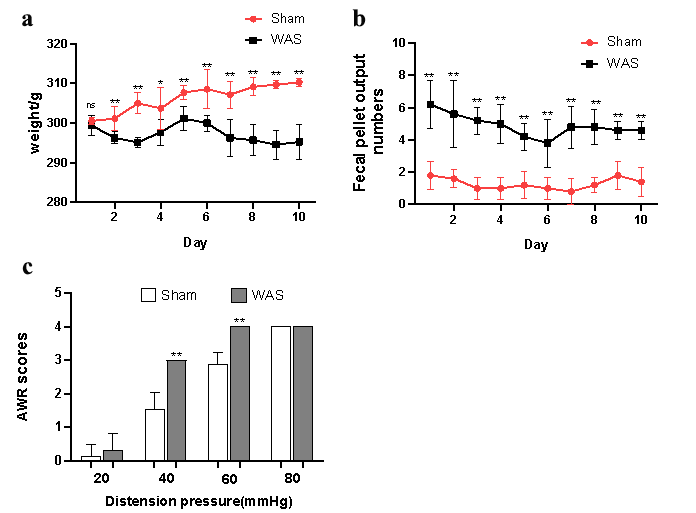
**

**Supplement. Figure 1** Effects of chronic water avoidance stress on body weight, fecal pellet output and visceral sensitivity in rats.

1. Changes of body weight in rats during chronic water avoidance stress. n=7/group. B. FPO number in the container after each 1-h WAS. n = 7/group. c. AWR scores in response to CRD. n = 7/group. **P* < 0.05, ***P* < 0.01.
