# Supplementary material for: Enteric glial cells contribute to chronic stress-induced alterations in the intestinal microbiota and barrier in rats
Source: Heliyon. 2024 Jan 23;10(3):e24899. doi: 10.1016/j.heliyon.2024.e24899 (PMC10838753; doi:10.1016/j.heliyon.2024.e24899)
Supplement: Multimedia component 2 [file mmc2.ppt]

## Slide 1
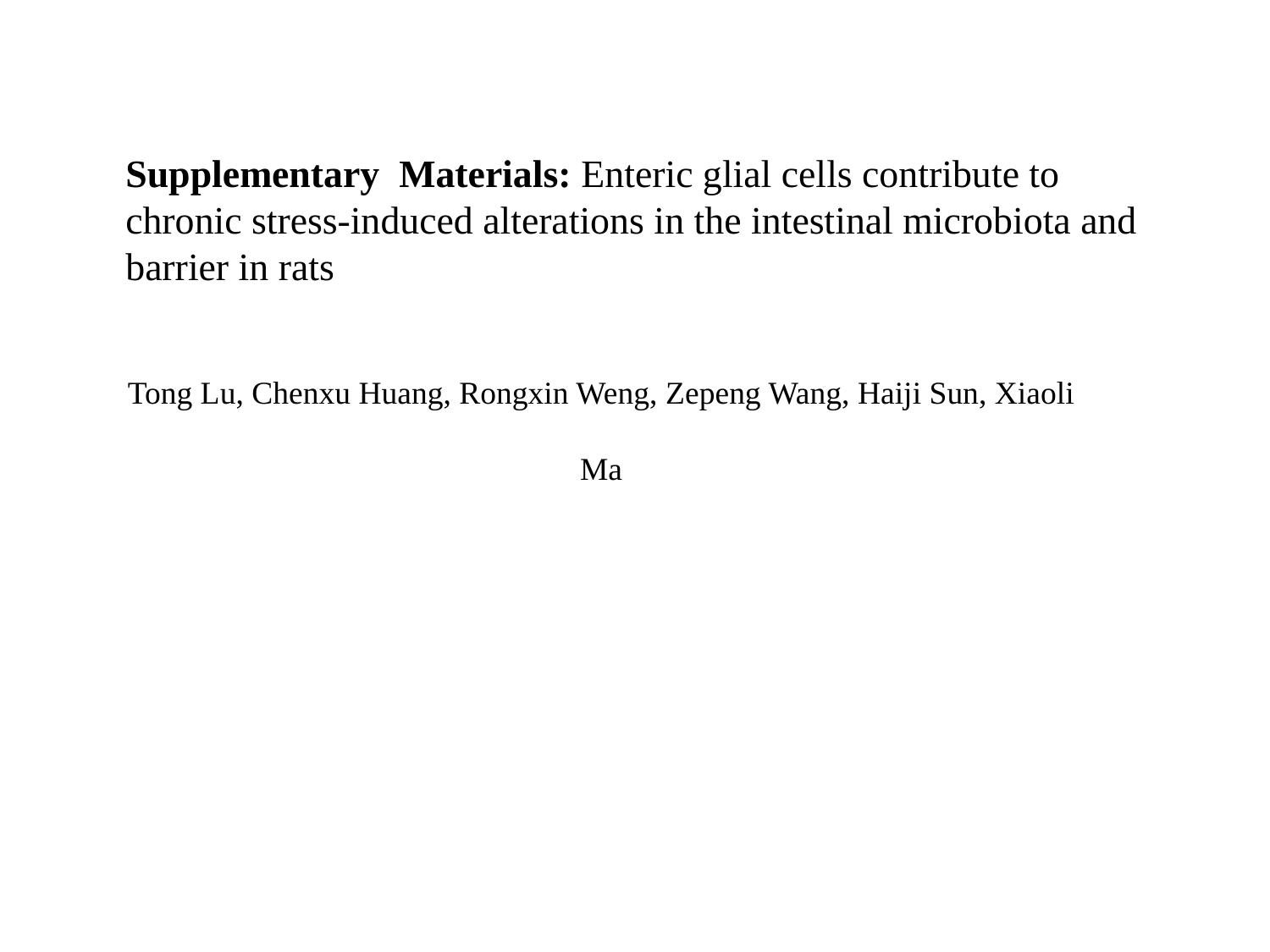

Supplementary Materials: Enteric glial cells contribute to chronic stress-induced alterations in the intestinal microbiota and barrier in rats
Tong Lu, Chenxu Huang, Rongxin Weng, Zepeng Wang, Haiji Sun, Xiaoli Ma

## Slide 2
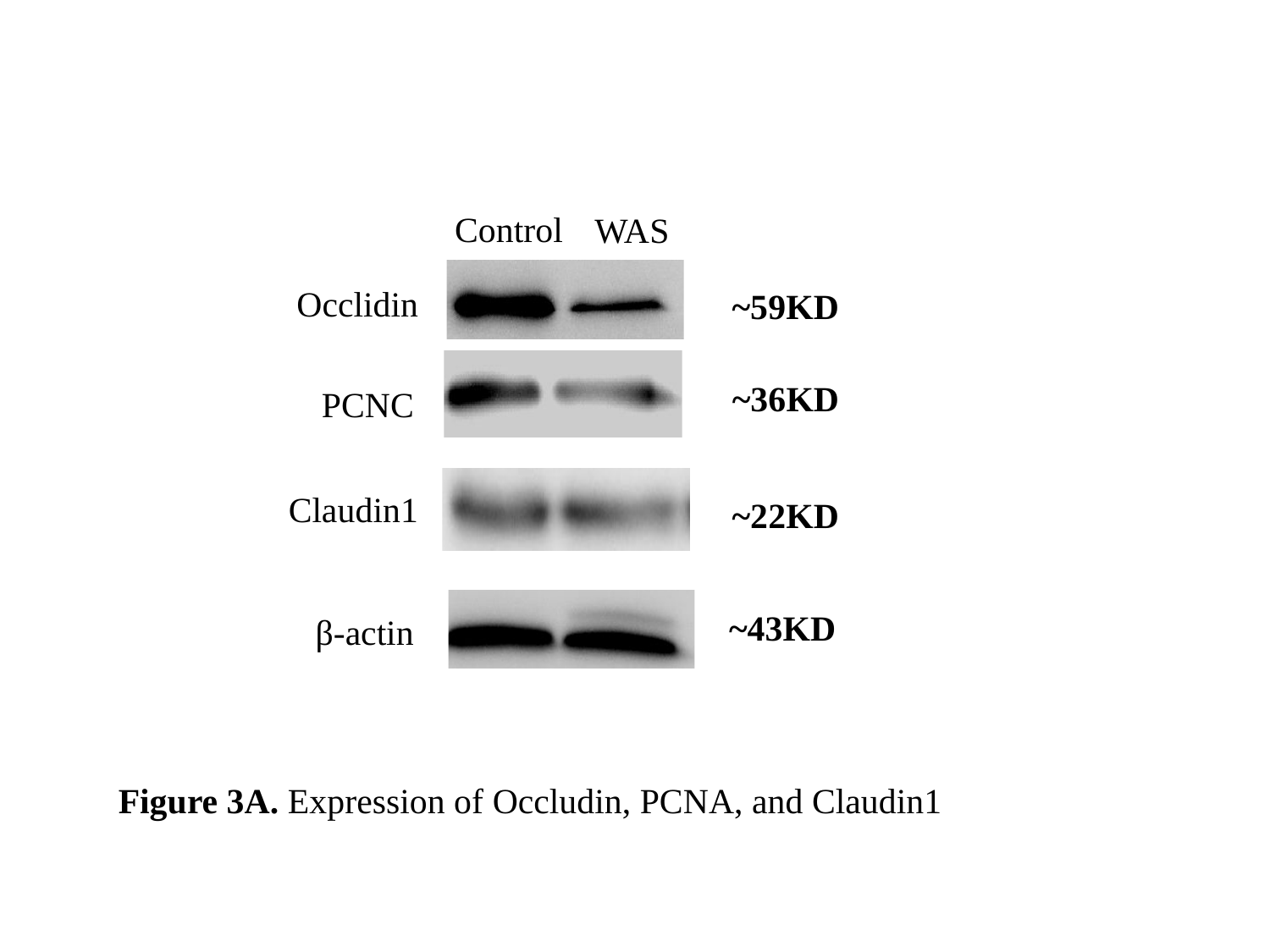

Control
WAS
Occlidin
~59KD
~36KD
PCNC
Claudin1
~22KD
~43KD
β-actin
Figure 3A. Expression of Occludin, PCNA, and Claudin1

## Slide 3
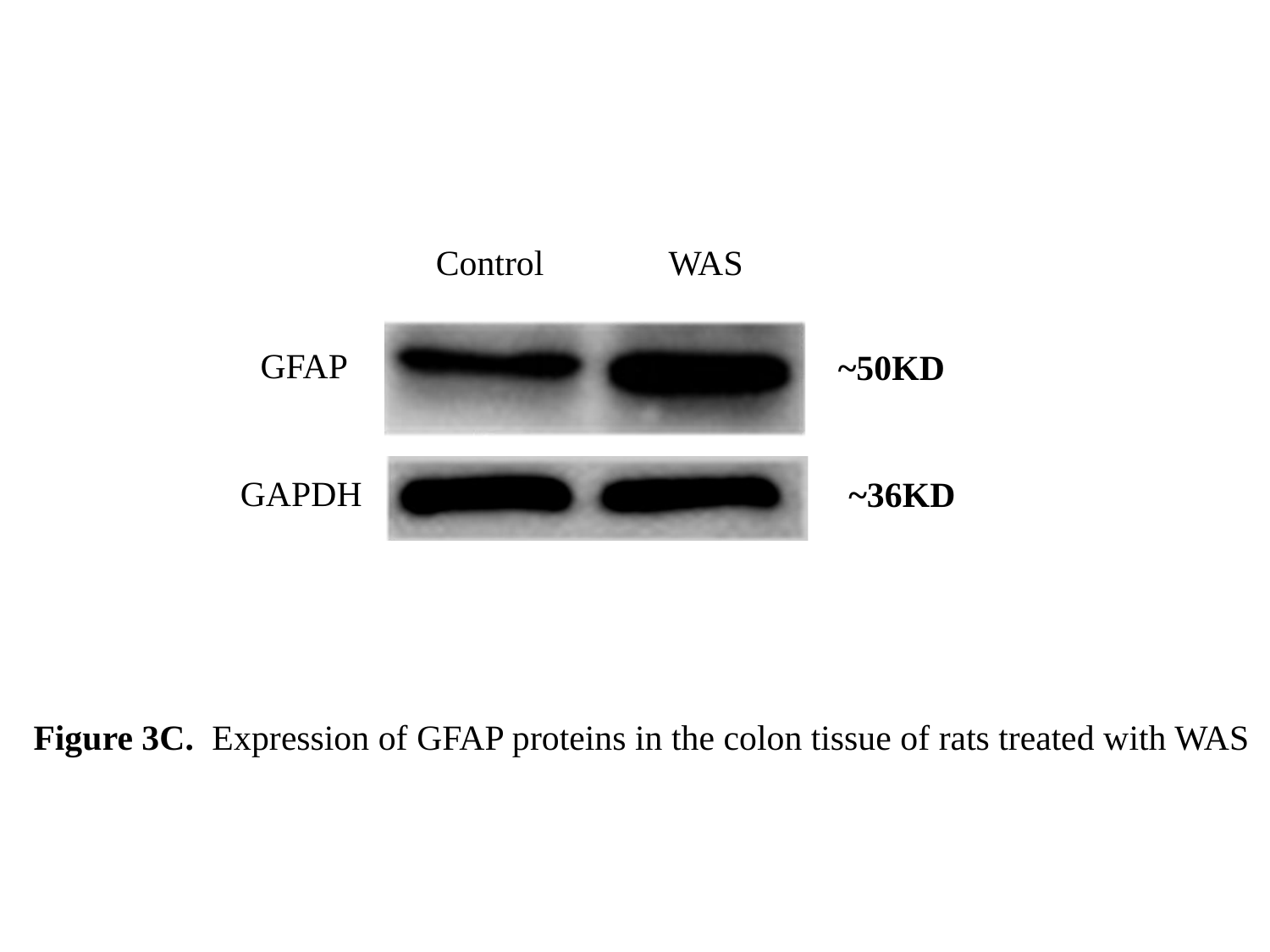

Control
WAS
GFAP
~50KD
GAPDH
~36KD
Figure 3C. Expression of GFAP proteins in the colon tissue of rats treated with WAS

## Slide 4
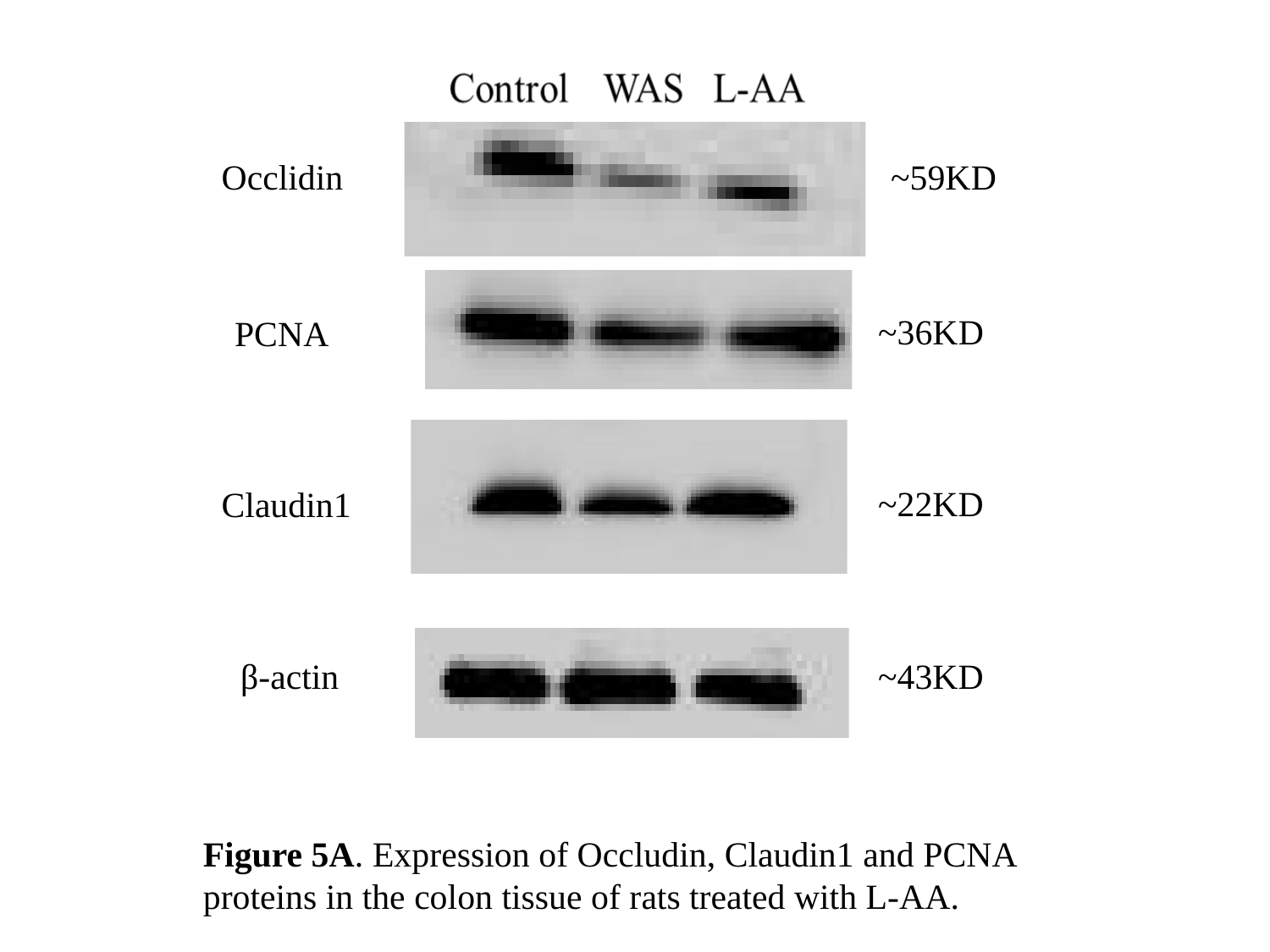

Occlidin
~59KD
~36KD
PCNA
~22KD
Claudin1
~43KD
β-actin
Figure 5A. Expression of Occludin, Claudin1 and PCNA proteins in the colon tissue of rats treated with L-AA.

## Slide 5
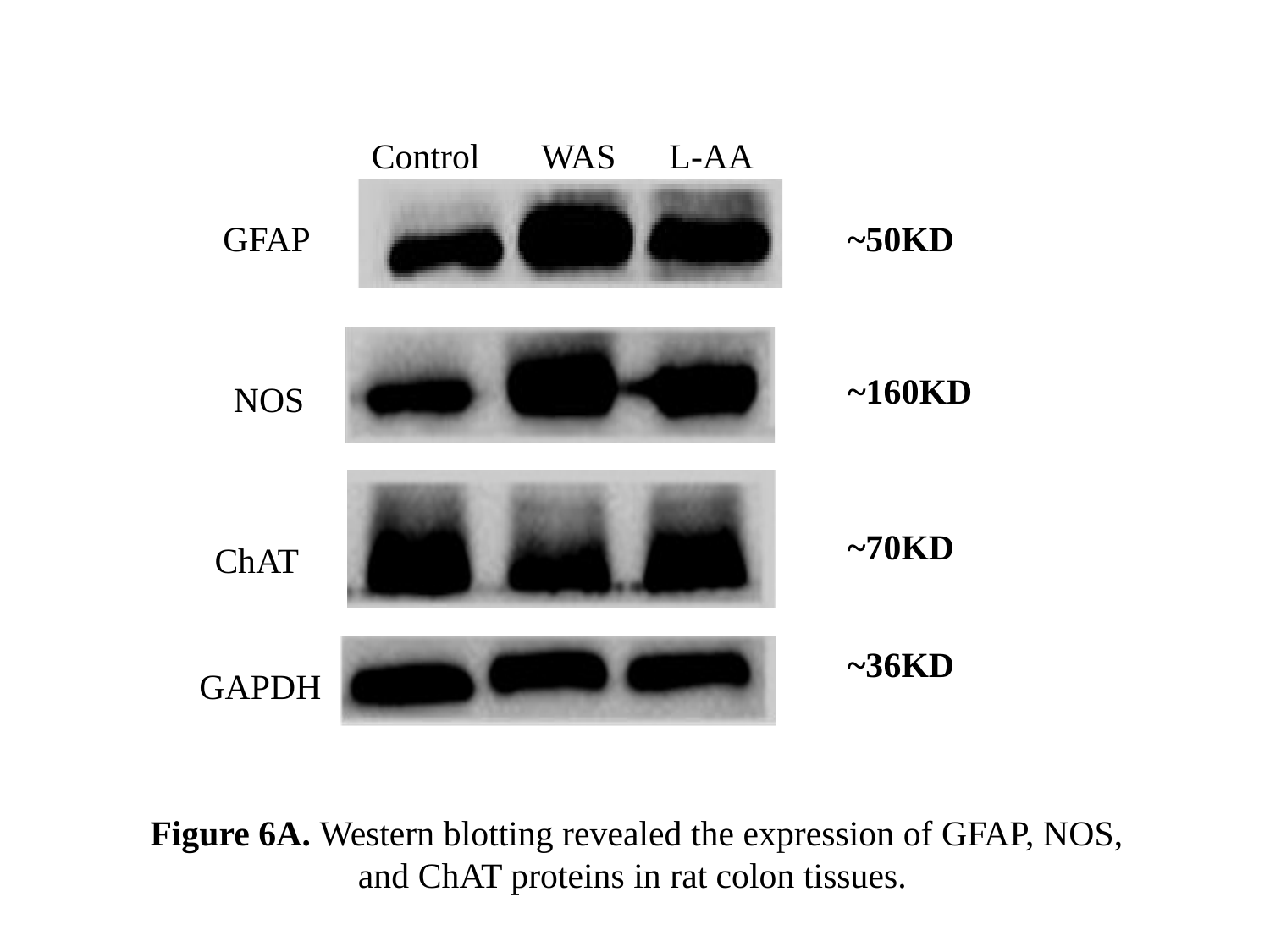

Control WAS L-AA
GFAP
~50KD
~160KD
NOS
~70KD
ChAT
~36KD
GAPDH
Figure 6A. Western blotting revealed the expression of GFAP, NOS, and ChAT proteins in rat colon tissues.
